# Comparison of laboratory costs of rapid molecular tests and conventional diagnostics for detection of tuberculosis and drug-resistant tuberculosis in South Africa

**DOI:** 10.1186/1471-2334-13-352

**Published:** 2013-07-29

**Authors:** Maunank Shah, Violet Chihota, Gerrit Coetzee, Gavin Churchyard, Susan E Dorman

**Affiliations:** 1 Johns Hopkins University School of Medicine, Department of Medicine, Division of Infectious Disease, 1503 East Jefferson St, Room 118, Baltimore, MD 21231, USA; 2 The Aurum Institute for Health Research, Johannesburg, South Africa; 3 National Health Laboratory Services, Johannesburg, South Africa; 4 London School of Hygiene and Tropical Medicine, London, UK; 5 School of Public Health, University of Witwatersrand, Johannesburg, South Africa

**Keywords:** Laboratory, Costs, Tuberculosis, Diagnostics

## Abstract

**Background:**

The World Health Organization has endorsed the use of molecular methods for the detection of TB and drug-resistant TB as a rapid alternative to culture-based systems. In South Africa, the Xpert MTB/Rif assay and the GenoType MTBDR*plus* have been implemented into reference laboratories for diagnosis of TB and drug-resistance, but their costs have not been fully elucidated.

**Methods:**

We conducted a detailed reference laboratory cost analysis of new rapid molecular assays (Xpert and MTBDR*plus*) for tuberculosis testing and drug-resistance testing in South Africa, and compared with the costs of conventional approaches involving sputum microscopy, liquid mycobacterial culture, and phenotypic drug sensitivity testing.

**Results:**

From a laboratory perspective, Xpert MTB/RIF cost $14.93/sample and the MTBDR*plus* line probe assay cost $23.46/sample, compared to $16.88/sample using conventional automated liquid culture-based methods. Laboratory costs of Xpert and MTBDR*plus* were most influenced by cost of consumables (60-80%).

**Conclusions:**

At current public sector pricing, Xpert MTB/RIF and MTBDR*plus* are comparable in cost to mycobacterial culture and conventional drug sensitivity testing. Overall, reference laboratories must balance costs with performance characteristics and the need for rapid results.

## Background

Currently, less than 10% of multi-drug resistant tuberculosis (MDR-TB) cases in the world are detected [1]. Performance of drug susceptibility testing (DST) using conventional methods relies on solid or liquid media and is slow and resource intensive. Recently, the World Health Organization endorsed the use of molecular methods for the detection of TB and drug-resistvant TB as a rapid alternative to culture-based systems [2,3]. Two commercially available molecular assays using different methodologies have been implemented in South Africa -- the GenoType MTBDR*plus* (‘MTBDRplus,’ Hain Lifescience, Nehren, Germany) and the Xpert MTB/RIF (‘Xpert,’ Cepheid, Sunnyvale, CA).

MTBDR*plus* is a line probe assay that has shown good diagnostic accuracy for the detection of *M. tuberculosis* in smear-positive cases and isoniazid and/or rifampin resistance in several validation studies, and test results can be available in as few as 1–2 days [4-6]. Xpert is an integrated specimen processing and nucleic acid amplification-based test for detection of *M. tuberculosis* and rifampin resistance and offers results within hours. Xpert has the advantage of high sensitivity when performed on smear microscopy-positive sputum samples and requires little laboratory processing, overhead, or labor [7].

South Africa is a middle-income country that has sought to scale-up laboratory services to implement these new tests. However, while Xpert and MTBDR*plus* offer rapid results and similar performance characteristics, each has limitations and neither may be a complete replacement for conventional culture and DST [2,3,6,7]. Both tests have reduced sensitivity for smear-negative samples, and conventional DST is needed for expanded drug resistance testing. MTBDR*plus* is a technically complicated assay requiring substantial laboratory resources, and both tests require expensive equipment and reagents. In August 2012, however, public sector prices for Xpert consumables were significantly reduced [8]. To date, little data is available to compare the costs and resource needs of these emerging rapid diagnostics to guide policy-makers and laboratory managers. To address this knowledge gap, we performed a detailed cost-analysis from a laboratory perspective and compared the costs associated with conventional liquid culture and DST, MTBDR*plus* and Xpert. We further explored the costs of incorporating these assays as stand-alone tests for TB diagnosis, or alternatively in conjunction with existing diagnostics.

## Methods

Costs associated with mycobacterial testing were analyzed from a laboratory perspective at the National Health Laboratory Services (NHLS) National TB Reference Laboratory in Johannesburg, South Africa. Costs were collected for testing conducted using Xpert, MTBDR*plus*, Ziehl-Neelsen smear microscopy using a light microscope, and florescence smear microscopy using auramine-O staining and a light emitting diode microscope [9]. Costs were also collected for sputum processing (i.e. digestion and decontamination) using N-acetyl-L-cysteine-NaOH and concentration by centrifugation [10], liquid culture using the automated BACTEC MGIT 960 system (BD Diagnostic Systems, Sparks, MD, USA), indirect phenotypic DST using the MGIT SIRE system (BD Diagnostic Systems), and anti-MPB64 monoclonal antibody-based species identification (Capilia TB-Neo, TAUNS Laboratories, Numazu, Japan) of positive cultures. For the liquid culture testing scenario, all samples were considered to require sputum processing and MGIT culture; positive cultures were tested by Ziehl Neelsen smear microscopy to assess for mycobacteria; cultures positive for mycobacteria were tested by the anti-MPB64 assay to distinguish *M. tuberculosis* from non-tuberculous mycobacteria, and cultures positive for *M. tuberculosis* were subjected to phenotypic MGIT SIRE DST. Xpert testing was conducted per manufacturer instructions and performed using a 4-module GeneXpert (Cepheid) instrument with automated readout. MTBDR*plus* was performed according to manufacturer instructions and consisted of DNA extraction, amplification, and hybridization steps, with hybridization performed using a GT Blot instrument (Hain Lifescience).

Costs were analyzed using an “ingredients” approach that involved multiplying the quantity of inputs used by their unit prices; costs and wages were gathered using detailed laboratory records. The amount of staff time, consumable supplies and equipment quantities utilized for each test were determined through direct observation of testing procedures, and included costs associated with quality assurance and quality control. Overhead laboratory costs included indirect labor costs, office and lab supplies and furniture, general operations costs, and physical infrastructure costs (i.e. building, utilities, and maintenance costs). Overhead costs were allocated based on the volume of testing and amount of physical infrastructure utilized by each diagnostic system. Equipment costs were annualized over their useful lifespans. South Africa is eligible to pay prices negotiated by the Foundation for Innovative Diagnostics (FIND), and costs for consumables and equipment reflect this pricing structure [8,11,12]. Laboratory testing capacity was estimated based on the laboratory operating for 12 hours per day. Ten percent of all sputum samples were estimated to be smear-positive and 5% of cultures were estimated to be positive for *M. tuberculosis* based on laboratory records. Unit costs of key equipment and consumables are shown in Table 1. We evaluated the costs of each diagnostic system individually and in combination with each other. All costs are presented in 2012 US dollars.

**Table 1:** Unit costs of key consumables and equipment*

**Consumables**	**Unit (quantity)**	**Unit cost $US**
NALC/NAOH Kit	per Sample	$1.73
N95 Mask	per Box(100)	$55.19
ZN stain	per liter	$4.87
Auramine-O	per liter	$4.67
Methylene blue	per liter	$3.26
Potassium permanganate	Per liter	$3.22
AFB fixative	per 100 ml	$8.52
PANTA	one box(100)	$89.07
MGIT growth supplement	one box(100)	$70.80
Anti-MPB64 Capilia TB Neo	per test	$1.69
MGIT Tube	per Box(100)	$195.00
SIRE kit	per kit (35)	$127.50
MTBDR rif kit	per kit (96)	$917.42
Xpert MTB/Rif cartridge	per cartridge	$9.98
**Equipment**		
Centrifuge plus accessories	per Instrument	$22,439
Vortex	per Instrument	$304
Biosafety cabinet	per Instrument	$3,190
Biosafety cabinet filter replacement	yearly	$1,608
Biosafety cabinet decontamination	q 6months	$156
Light microscope	per Instrument	$3,409
LED microscope	per Instrument	$4580
Bactec MGIT 960	per Instrument	$38,950.00
Epicenter software	--	$12,500.00
Barcode scanner	per Instrument	$158.30
Thermocycler	per Instrument	$5,621.18
Ultrasonicator	per Instrument	$1,596.42
GTBLOT maintenance	semi-annual	$487.08
GT BLOT	per Instrument	$17,557.40
UPS (power supply)	per Instrument	$137.24
Xpert instrument	per Instrument	$17,000.00
Xpert calibration	per 1800 runs	$1,800.00

*Not all items are shown. Additional minor consumables and equipment costs included but were not limited to costs associated with gloves, disposable gowns, pipettes and pipetters and tips, computers and supplies, soap and disinfectant, waste disposal including biohazard bags, microscopy slides, and other common microbiology supplies.

## Results

Base-case laboratory costs for TB diagnostics are shown in Table 2. Costs per test conducted were $14.93 for Xpert, $23.46 for MTBDR*plus,* $3.40 for fluorescence smear microscopy, $2.25 for Ziehl-Neelson light microscopy, $12.16 for MGIT culture, and $26.19 for DST using MGIT SIRE. We calculated a total cost of $16.88 per specimen tested for the combination of fluorescence smear microscopy plus the liquid culture testing scenario (sputum processing and MGIT culture, followed by Ziehl Neelsen smear microscopy on positive cultures, anti-MPB64 assays on cultures with mycobacterial growth, and MGIT SIRE DST on cultures with growth of *M. tuberculosis*). This combination of fluorescence microscopy plus the liquid culture testing scenario was over $6 cheaper per sample than using MTBDR*plus* on all sputa, but was more expensive than Xpert (Table 3).

**Table 2:** Component costs for each tuberculosis diagnostic test

	**Consumables cost per test, in $ (% of total) [uncertainty range]**^**†**^	**Equipment cost per test, in $ (% of total) [ uncertainty range]**^**†**^	Labor * cost per test, in $ (% of total) [uncertainty range]^**†**^	**Overhead cost per test, in $ (% of total) [uncertainty range]**^**†**^	**Total cost per test, in $ [uncertainty range]**^**†**^
**Fluorescence smear microscopy**	$0.36 (10%) [$0.27–$0.45]	$0.12 (4%) [$0.09–$0.48]	$2.18 (64%) [$1.64–$3.00]	$0.74 (22%) [$0.18–$0.93]	$3.40 [$2.19–$4.76]
**Ziehl-Neelsen light smear microscopy**	$0.34 (15%) [$0.26–$0.43]	$0.11 (5%) [$0.08–$0.45]	$1.05 (47%) [$0.88–$1.53]	$0.74 (33%) [$0.18–$0.93]	$2.25 [$1.40–$3.24]
**MGIT culture**	$8.05 (66%) [$6.04–$8.65]	$1.05 (9%) [$0.71–$4.74]	$2.17 (18%) [$2.05–$2.50]	$0.89 (7%) [$0.66–$1.11]	$12.16 [$9.46–$16.99]
**Phenotypic DST using MGIT SIRE system**	$16.22 (61%) [$12.16–$18.71]	$2.77 (10%) [$2.08–$13.42]	$4.15 (16%) [$3.41–$6.16]	$3.26 (12%) [$2.45–$4.07]	$26.39 [$20.10–$42.37]
**MTBDR*plus***	$14.13 (60%)[$10.37–$17.24]	$1.60 (7%) [$4.17–$7.39]	$3.46 (15%) [$2.85–$5.13]	$4.28 (18%) [$3.21–$5.35]	$23.46[$20.61–$35.12]
**Xpert MTB/RIF**	$11.97 (80%) [$11.49–$19.47]	$0.93 (6%) [$0.70–$3.99]	$1.13 (8%) [$0.94–$4.30]	$0.90 (6%) [$0.22–$1.12]	$14.93 [$13.36–$28.88]

*Abbreviations*: *MGIT* Mycobacterial Growth Indicator 960 automated liquid culture system, *DST* Drug Susceptibility Testing using MGIT SIRE system.

*Average salary for laboratory technician was $9.07 per hour based on laboratory records. Range of wages from $7.43 to $16.16 were used for sensitivity analysis based on wages of different skill levels of laboratory workers.

^†^Uncertainty range is based on lowest and highest estimates of consumable and equipment components along with range of laboratory volume of testing [e.g. batch size of Xpert testing was varied from 1 sample to 4 samples per run], along with range of salaries for laboratory technicians, and highest and lowest estimates for laboratory overhead.

**Table 3:** Expected costs of diagnostic algorithms

**Algorithm**	**Cost per sample**	**Incremental cost**
**Implementation of conventional diagnostics versus molecular testing**		
Fluorescence microscopy plus liquid culture testing scenario* on all sputa	$16.88	reference
Xpert MTB/RIF alone on all sputa	$14.93	$-1.95
MTBDR*plus* alone on all sputa	$23.46	$6.58
**Intensive implementation of molecular tests in combination with conventional diagnostics**		
Xpert MTB/RIF plus liquid culture testing scenario* on all sputa	$28.41	$11.53
MTBDR*plus* plus liquid culture testing scenario* on all sputa	$36.94	$20.06
**Conventional diagnostics with selective implementation of molecular tests**		
Fluorescence microscopy plus liquid culture testing scenario* on all sputa + Xpert MTB/RIF on smear-positive sputa	$18.37	$1.49
Fluorescence microscopy plus liquid culture testing scenario* on all sputa + MTBDR*plus* on smear-positive sputa	$19.23	$2.35
Fluorescence microscopy on all sputa plus a) Xpert MTB/RIF on smear-positive sputa and b) liquid culture on smear-negative sputa with Xpert MTB/RIF on culture positive isolates	$16.51	-$0.37
Fluorescence microscopy on all sputa plus a) MTBDR*plus* on smear-positive sputa and b) liquid culture on smear-negative sputa with MTBDR*plus* on culture positive isolates	$17.75	$0.87
Molecular testing with selective implementation of culture and dst**		
Xpert MTB/RIF on all sputa, with liquid culture testing scenario* on sputa with a positive molecular test	$16.86	$-0.02
MTBDR*plus* on all sputa, with liquid culture testing scenario* on sputa with a positive molecular test	$25.39	$8.51

*Abbreviations*: *MGIT* Mycobacterial Growth Indicator 960 automated liquid culture system, *DST* Drug Susceptibility Testing using MGIT SIRE system.

* Liquid culture testing scenario consists of sputum processing and MGIT culture, followed by Ziehl Neelsen smear microscopy on positive cultures, anti-MPB64 assays on cultures with mycobacterial growth, and MGIT SIRE DST on cultures with growth of *M. tuberculosis.*

**Utilizes current estimates of Xpert and MTBDR*plus* sensitivity and specificity and prevalence of TB and drug-resistant TB in South Africa.

The cost of Xpert was largely attributable to consumables ($11.97 per test [80% of total]), and was driven by the cost of Xpert cartridges ($9.98 per cartridge, Tables 1 and 2). Alternatively, countries not eligible for FIND-negotiated discounts may pay up to $78,200 for purchase of the GeneXpert instrument and $71.63 per cartridge [8,13]. In the latter scenario, Xpert costs may rise to $78.94 per sample and would be substantially more expensive than conventional diagnostics. Labor costs associated with performing Xpert were low ($1.13 per test) compared to MGIT culture ($2.17 per test) or MTBDR*plus*($3.46 per test). Overall, if the volume of testing in the laboratory were reduced by 50%, the cost of Xpert would rise to $16.50 per test.

By comparison, MTBDR*plus* costs were similarly attributable largely to consumables ($14.13 per test [60%]), but also had high labor costs ($3.46 per test) and overhead costs ($4.28 per test) due to the time involved and extensive laboratory facilities needed for test performance.

Laboratory costs associated with alternative diagnostic algorithms incorporating multiple tests are shown in Table 3. An intensive TB diagnostic strategy involving performance of Xpert or MTBDR*plus* in addition to the liquid culture testing scenario on all sputum samples would nearly double laboratory costs per sample ($28.41 and $36.94 per sample for addition of Xpert and addition of MTBDR*plus*, respectively) compared to a strategy of using only the liquid culture testing scenario (Table 3).

A more selective strategy considered by some laboratories to reduce costs might be to perform smear microscopy plus the liquid culture testing scenario on all sputum samples to ensure highest diagnostic sensitivity, but employ molecular testing only selectively for smear microscopy-positive samples (for MTB confirmation and rapid drug-resistance results). This strategy would lead to an incremental cost of less than $3 per sample, compared to using smear microscopy plus the liquid culture testing scenario (Table 3), while likely substantially reducing the time to diagnosis of resistance.

An additional strategy considered by some labs may be to utilize molecular assays primarily as a replacement for conventional DST. Such a strategy would cost $16.51 if Xpert were used in place of DST (incremental -$0.37 compared to liquid culture scenario with conventional DST) and $17.75 if MTBDR*plus* were used in place of DST (incremental $0.87 compared to liquid culture scenario with conventional DST).

In South Africa, recent guidelines suggest using GeneXpert for all high risk patients and to additionally perform conventional culture and DST for confirmation of positive molecular test results [14]. Such a strategy would cost $16.86 per TB suspect using GeneXpert as the molecular assay and $25.39 if MTBDR*plus* were used (Table 3).

## Discussion

Scale-up of laboratory capacity for detection of TB and drug resistance is urgently needed, but may be costly. Current reference standard approaches involving mycobacterial culture and DST are slow, and are resource intensive for laboratories to perform. The Xpert MTB/Rif and MTBDR*plus* are two WHO recommended platforms for rapid detection of TB and drug-resistant TB and many low and middle-income countries qualify for negotiated discounts on these assays [8]. Previously, there has been limited cost information from a laboratory perspective to guide TB control programs and laboratories in implementing these tests. Our results suggest that with recent reductions in the price of Xpert cartridges, the cost of Xpert testing is comparable to that of conventional diagnostics, making it possible to consider replacement of sputum microscopy and culture with Xpert from a laboratory cost standpoint. We found that the cost of Xpert testing ($14.93) in a reference laboratory in South Africa was similar to performing the current reference standard of smear-microscopy followed by liquid culture and conventional DST ($16.88); MTBDR*plus* was found to be the most costly ($23.46) but offers the benefit of rapid isoniazid resistance testing in addition to rifampin resistance testing. Costs of molecular testing were most influenced by consumable costs which accounted for 60-80% of total costs associated with Xpert and MRTBD*plus.* Xpert additionally offers the benefit of reduced staff time needed for testing, with potential to increase volume of testing or increased opportunities and time to perform other diagnostic tests or laboratory activities.

Overall, laboratories and TB control programs must balance costs with performance characteristics and the need for rapid results [15]. Both molecular tests assessed in this study offered rapid detection of *M. tuberculosis* and drug-resistance. However, reliance on either Xpert or MTBDR*plus* alone for diagnosis of TB and drug-resistant TB in place of liquid culture and DST has limitations. Each has suboptimal sensitivity in individuals with smear-negative TB, and neither allows testing of second line drugs; Xpert also does not allow assessment of isoniazid mono-resistance [6,7]. To maximize detection of *M. tuberculosis* and drug-resistance, laboratories may choose to perform conventional culture and DST in addition to newer molecular assays. We found that intensive implementation of molecular testing in conjunction with conventional diagnostics for all sputa in order to optimize both sensitivity and speed of diagnosis would lead to significant laboratory cost increases (70-120% increase), making this option potentially unaffordable for laboratories in some settings.

To assist laboratories in allocation of resources, we examined the costs of multiple diagnostic algorithms. Alternative diagnostic algorithms with selective application of molecular assays may be considered in some laboratories. We found that selectively performing Xpert or MTBDR*plus* for smear-positive samples (while additionally performing liquid culture and DST for all samples) leads to only modest increases in laboratory costs (incremental $1.49 and $2.35 per sample for Xpert and MTBDR*plus*, respectively). Such a strategy would allow rapid species-level assessment of *M. tuberculosis* and rapid identification of drug-resistant TB in those likely to be most infectious, while also allowing performance of reference standard testing on all patients. Similarly, we found only relatively small increases to laboratory costs if molecular testing were used as a rapid alternative to conventional indirect phenotypic DST.

Our study has several limitations. Not all countries qualify for negotiated reduced pricing, and costs associated with labor (i.e. wages) and overhead can vary geographically. Nonetheless we provide a detailed cost-breakdown to aid generalizability and allow laboratories in other settings to estimate costs associated with implementing these emerging rapid TB diagnostic tests and provide cost estimates for Xpert testing without negotiated prices. This study was a cost-analysis from a reference laboratory perspective. Cost of transportation to a reference laboratory were not included in this analysis and may represent a significant expense and vary geographically; alternatively, implementation of Xpert at a lower level of the health system may avert specimen transport costs associated with mycobacterial culture and conventional DST that must be performed in more established laboratories. TB control programs making decisions on diagnostic algorithms must additionally consider costs associated with clinical evaluation and TB treatment in addition to laboratory costs, as well as consider the local prevalence of TB and drug-resistance and the need for rapid diagnosis. Nevertheless, our study provides important information regarding the likely diagnostic costs associated with incorporating Xpert and MTBDR*plus* as part of future diagnostic strategies.

On the other hand, our study has several strengths. We incorporated recent negotiated price reductions to aid generalizability to other low and middle-income settings, and report the component of costs attributable to consumables, equipment, and labor for each TB diagnostic system. We found that the Xpert and MTBDR*plus* laboratory costs are comparable to those of conventional diagnostics and should be considered as part of TB diagnostic algorithms; we additionally offer insight into the costs of alternative algorithms that some laboratories may be considering. Our results provide important information to aid future studies evaluating cost-effectiveness and implementation of emerging TB diagnostic algorithms and TB case-finding strategies.

## Conclusions

The cost of newer molecular diagnostic tests are comparable to conventional diagnostic methods, when paying reduced negotiated pricing for Xpert and MTBDR*plus.* We present detailed cost information related to implementation of these rapid molecular assays to guide laboratories seeking to scale up TB diagnostics. Overall, laboratories and TB programs must balance costs with performance characteristics and the need for rapid results. Intensive implementation of molecular assays as an addition to conventional automated liquid culture and DST may lead to significant laboratory cost increases; selective implementation of molecular assays could be considered for some settings.

## Competing interests

The authors declare that they have no competing interests.

## Authors’ contributions

MS conducted data collection, data analysis, and led manuscript writing and study design. VC, GC, GC assisted with data collection and manuscript writing. SD assisted with manuscript writing, study design, and conceived the study. All authors read and approved the final manuscript.

## Pre-publication history

The pre-publication history for this paper can be accessed here:

http://www.biomedcentral.com/1471-2334/13/352/prepub
